# Advanced Skeletal Muscle Mass Reduction (Sarcopenia) Secondary to Neuromuscular Disease

**DOI:** 10.1155/2020/8834542

**Published:** 2020-07-11

**Authors:** G. R. Pesola, V. Terla, M. Pradhan

**Affiliations:** ^1^Department of Epidemiology, Mailman School of Public Health, Columbia University, New York, NY, USA; ^2^Department of Medicine, Section of Critical Care/Pulmonary Medicine, Harlem Hospital/Columbia University, New York, NY, USA; ^3^Department of Critical Care Medicine, Memorial Sloan-Kettering Cancer Center, New York, NY, USA; ^4^Department of Medicine, Jacobi Hospital, Bronx, NY, USA

## Abstract

We describe a young male patient chronically on a ventilator secondary to decreased mobility from amyotrophic lateral sclerosis (ALS). He had both a tracheostomy for breathing and percutaneous endoscopic gastrostomy (PEG) for feeding. Using 24-hour urinary creatinine excretion data, we calculated an estimate of skeletal muscle (SM) mass. SM mass was indexed to height and weight to obtain the SM index. The SM index is used as a determinant to define sarcopenia. From the data, we found that this patient had the smallest SM index ever recorded at 2.2 kg/m^2^, consistent with extremely advanced sarcopenia. As a comparison, “severe” sarcopenia in a male is defined as a SM index ≤ 8.5 kg/m^2^. This method can be used in ICU patients to evaluate for sarcopenia which is a predictive marker for mortality.

## 1. Introduction

**Figure 1:**
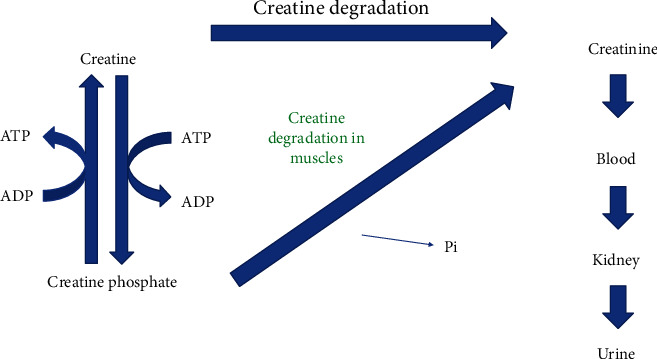
Diagram reveals constant nonenzymatic degradation of muscle creatine to creatinine. This occurs at a constant rate of 1.5 to 2% of skeletal muscle per day. Daily urinary creatinine excretion is directly proportional to an individual's muscle mass.

Almost all of body creatine is found in the skeletal muscle which is converted nonenzymatically at a constant rate to serum creatinine (see Figure 1). With normal renal function, the only route of creatinine elimination is the kidney. Renal creatinine excretion is constant and directly proportional to muscle mass at steady state (a constant serum creatinine). Therefore, daily urinary creatinine excretion is a good estimate of muscle mass [1].

Creatinine excretion is highly correlated with lean body mass [2] and in theory is a better estimate of muscle mass than lean body mass since creatinine is derived from creatine in muscle [3]. It is felt that the creatine content of muscle does not change with age less than 60 [4], making a 24 hr collection of urinary creatinine a good estimate of muscle mass. It is inexpensive, a direct estimate, and valid at younger ages.

Normal creatinine excretion in healthy subjects tends to be 15 mg/kg/day or higher, but it has been described below 10 mg/kg/day in elderly critically ill ICU patients and as low as 6 mg/kg/day [5]. The purpose of this study was to determine SM mass, as estimated by urinary creatinine excretion [1], in a critically ill patient who had been bedridden and unable to move for several years.

## 2. Materials and Methods

Demographic data and two 24 hr urine collections done as part of routine care to estimate creatinine clearance [6] were retrospectively evaluated. The 24 hr urine collections are only done in patients with a creatinine less than 2 mg/dl and at steady state defined as unchanging plasma creatinine levels during collection.

Estimation of SM mass (kilograms (kg)) was determined using the 24-hr urine creatinine collection with the formula: SM mass (kg) = 18.9 × Cr (grams) + 4.1 [1]. Calculation of the SM index is done by substituting patient height and SM mass (kg) into the BMI formula. The formula for ideal body weight was taken from the ARDSNET trial [7].

Body mass index (BMI) is defined as weight divided by height squared: [mass (kg)/height (m)^2^].

The coefficient of variation is the S.D. divided by the mean and is often expressed as a percent.

Skeletal muscle mass is defined in this article as the estimated mass (kg) of SM obtained from creatinine excretion over 24 hours [1]. When SM mass is adjusted for height and weight [8, 9], it will be called SM index, albeit these terms can be loosely used interchangeably.

Normal SM mass in men has been defined as ≥10.76 kg/m^2^ [8]. Severe sarcopenia has been defined as a SM mass in men of ≤8.5 kg/m^2^ [8]. This SM mass expression has also been called the SM index in some literature [9]. With aging, the prevalence of primary sarcopenia has been shown to occur in 4.6% of men and 7.9% of women with a mean age of 67 [10]. In this case, the young patient was felt to have secondary sarcopenia due to a lack of mobility from ALS.

The Institutional Review Board from Harlem Hospital waived approval of this retrospective case of previously collected clinical and renal function data (Brany File # 19-15-187-273 (HHC)).

## 3. Case Presentation

A 35-year-old 105.5 kg male with a previous history of ALS for 7 years, history of respiratory insufficiency with a tracheostomy, and PEG for over two years, was admitted from a nursing home after a cardiac arrest. The return of spontaneous circulation (ROSC) was 12 minutes, and the patient was treated for aspiration pneumonia and urinary tract infection. In the past, he has had three previous cardiac arrests associated with respiratory insufficiency. Cardiac echocardiogram revealed a normal left and right ventricular function. Serum albumin was 3.1 g/dl. The patient has had quadriplegia felt secondary to his ALS and multiple cardiac arrests and has always stabilized and been treated for various infections. Neurologically, the patient still had a pupillary reflex on the left with spontaneous eye movement and he had partial mouth opening and closing. The patient does not visibly respond to pain. No gag or corneal reflexes were noted by the ICU team or neurology.

Over the last several years, the family was approached by the health care team on multiple occasions regarding consideration of Do Not Resuscitate (DNR). The family was told that the overall situation was hopeless due to his underlying primary disease. Despite this, the family always had hope that an ALS cure would be found and always wanted everything done.

Initially, a 24 hr urine creatinine was collected (patient with indwelling foley catheter) and was 172 mg. This was repeated since the value was very low but still came back 199 mg of urinary creatinine in 24 hr. The coefficient of variation was 10%, and the average was 186 mg of urinary creatinine excreted in 24 hr (see Table 1 for data summary). In addition, the patient's height was 185 cm.

The patient was treated successfully over several weeks in the ICU and was eventually transferred back to his nursing facility. Within one month after discharge, the patient died at another facility.

## 4. Discussion

Almost all of body creatine is found in the skeletal muscle which is converted nonenzymatically at a constant rate to serum creatinine (see Figure 1). With normal renal function, the only route of creatinine elimination is the kidney. Renal creatinine excretion is constant and directly proportional to muscle mass at steady state (a constant serum creatinine). Therefore, daily urinary creatinine excretion is a good estimate of muscle mass [1].

Urinary creatinine excretion has been shown to be an independent predictor of all-cause mortality in ambulatory adults; there was an inverse relationship between creatinine excretion and mortality over 13 years [11]. This mortality relationship may be related to reduced muscle mass as indirectly measured by 24 hr urinary creatinine excretion [11]. A second prospective study, in ambulatory adults aged 55 or older, measured SM mass using bioelectrical impedance. There was an inverse association between SM mass and all-cause mortality [9]. In two other prospective studies, those with sarcopenia as community dwellers relative to those without sarcopenia, there was a much higher mortality over time in those with sarcopenia [12, 13].

In ICU patient's, critical illness results in a very rapid loss of skeletal muscle in the first two to three weeks of severe illness [14, 15]. After a critical illness episode with survival, patients who then become bed-bound can lose up to 50% of SM mass within 4 months [16]. In a small case series of three, all patients developed a SM mass of less than 4 kg/m^2^ and all patients died [16]. In mechanically ventilated ICU patients who start with low SM mass at baseline, mortality is much higher than similar patients who have a normal SM mass [17]. Finally, a previous study in 149 elderly (age ≥ 65) surgical trauma ICU patients found that those with sarcopenia had decreased ventilator-free days (*p* < 0.025), ICU-free days, and higher all-cause mortality compared to those without sarcopenia [18]. All these studies suggest that reduced SM mass in the ICU setting is associated with increased mortality. These ICU studies are also consistent with studies of free-living adults [9, 11–13].

Our patient had reduced SM mass from the viewpoint of both a reduction in creatinine excretion at 1.76 mg/kg/day and SM index at 2.2 kg/m^2^. A previous study has revealed creatinine excretion of greater than 6 mg/kg/day with patient survival [5] and another study with creatinine excretion of 3.5 to 5.9 mg/kg/day without patient survival [16]. In this latter study, all patients had a SM index < 4 kg/m^2^ before they died [16].

Mechanisms for reduction in the SM mass in our patient (with advanced secondary sarcopenia) include recurrent sepsis, both aspiration pneumonia and urinary tract infections, that the patient has had over the years as well as the lack of muscle loading that maintains muscle mass. Advanced ALS with denervation of muscles and interruption of enteral nutritional support were probably other factors [8, 15].

What this study means is that there is a relatively easy way to determine “severe” sarcopenia in critically ill and even chronically ill patients in the ICU using the SM index [8]. When the SM index is ≤ 5.75 kg/m^2^ in women or ≤8.5 kg/m^2^ in men, these cut-offs define severe sarcopenia by muscle mass [8]. In addition to reduced SM index, sarcopenia also requires a measure of reduced strength (often measured by grip strength) or reduced performance (often measured by a gait speed ≤ 0.8 m/sec). In our patient or one other study with SM index < 4 kg/m^2^ [16], no patient was able to move, all consistent with advanced sarcopenia.

We agree with one point of view that there is still a very good reason to measure 24 hour urine creatinine excretion, to get an estimate of muscle mass [19]. In the ICU setting, severe sarcopenia will probably predict ICU morbidity and mortality if done in larger studies. In our patient and three other patients with a SM index less than 4 kg/m^2^ [16], all patients died in less than one month after detection of advanced sarcopenia.

## 5. Conclusions

Due to advanced ALS in this patient, we detected a SM index of 2.2 kg/m^2^. This value is so low it probably could only be found in a patient with advanced neurologic disease and has never been seen before. Using 24-hour urine collections, when possible, many ICU patients and even other chronically ill hospitalized patients can be risk stratified to those with and without “severe” sarcopenia. From there, all-cause mortality can evaluate overtime whether sarcopenia is a predictive difference.

## Figures and Tables

**Table 1:** Summary of creatinine excretion and SM mass calculations.

Patient (35 male with ALS)	Weight (kg)	BMI	24 hr urine creatinine (mg)	Creatinine (mg/kg/day)	SM mass (kg/m^2^)
Actual Wt.	105.5	30.8	186	1.76	2.2
I.D. Wt.	79.5	23.2		2.33	

SM: skeletal muscle; SM mass formula in kg = 18.9 × Cr (grams) + 4.1 [1]; I.D. Wt.: ideal body weight [7]. BMI: body mass index = [mass (kg)/height (m)^2^]; Actual Wt.: actual weight; severe sarcopenia in men: SM mass ≤ 8.5 kg/m^2^.

## Data Availability

All data is available in the manuscript.

## References

[1] Wang Z., Gallagher D., Nelson M. E., Matthews D. E., Heymsfield S. B. (1996). Total-body skeletal muscle mass: evaluation of 24-h urinary creatinine excretion by computerized axial tomography. *The American Journal of Clinical Nutrition*.

[2] Forbes G. B., Bruining G. J. (1976). Urinary creatinine excretion and lean body mass. *The American Journal of Clinical Nutrition*.

[3] Welle S., Thornton C., Totterman S., Forbes G. (1996). Utility of creatinine excretion in body-composition studies of healthy men and women older than 60 y. *The American Journal of Clinical Nutrition*.

[4] Forsberg A. M., Nilsson E., Werneman J., Bergström J., Hultman E. (1991). Muscle composition in relation to age and sex. *Clinical Science*.

[5] Pesola G. R., Akhavan I., Carlon G. C. (1993). Urinary creatinine excretion in the ICU: low excretion does not mean inadequate collection. *American Journal of Critical Care*.

[6] Pesola G. R., Akhavan I., Madu A., Shah N. K., Carlon G. C. (1993). Prediction equation estimates of creatinine clearance in the intensive care unit. *Intensive Care Medicine*.

[7] ARDS Network (2000). Ventilation with lower tidal volumes as compared with traditional tidal volumes for acute lung injury and the acute respiratory distress syndrome. *The New England Journal of Medicine*.

[8] Cruz-Jentoft A. J., Baeyens J. P., Bauer J. M. (2010). Sarcopenia: European consensus on definition and diagnosis: report of the European Working Group on Sarcopenia in Older People. *Age and Ageing*.

[9] Srikanthan P., Karlamangla A. S. (2014). Muscle mass index as a predictor of longevity in older adults. *The American Journal of Medicine*.

[10] Patel H. P., Syddall H. E., Jameson K. (2013). Prevalence of sarcopenia in community dwelling older people in the UK using the European Working Group on Sarcopenia in Older People (EWGSOP) definition: findings from the Hertfordshire Cohort study (HCS). *Age Aging*.

[11] Oterdoom L. H., Gansevoort R. T., Schouten J. P., de Jong P. E., Gans R. O. B., Bakker S. J. L. (2009). Urinary creatinine excretion, an indirect measure of muscle mass, is an independent predictor of cardiovascular disease and mortality in the general population. *Atherosclerosis*.

[12] Bigaard J., Frederiksen K., Tjønneland A. (2014). Body fat and fat-free mass and all-cause mortality. *Obesity Research*.

[13] Bunout D., de la Maza M. P., Barrera G., Leiva L., Hirsch S. (2011). Association between sarcopenia and mortality in healthy older people. *Australasian Journal on Ageing*.

[14] Gruther W., Benesch T., Zorn C. (2008). Muscle wasting in intensive care patients: ultrasound observation of the M. Quadriceps Femoris muscle layer. *Journal of Rehabilitation Medicine*.

[15] Puthucheary Z. A., Rawal J., McPhail M. (2013). Acute skeletal muscle wasting in critical illness. *JAMA*.

[16] Khan J., Bath K., Hafeez F., Kim G., Pesola G. R. (2018). Creatinine excretion as a determinant of accelerated skeletal muscle loss with critical illness. *Turkish Journal of Anesthesia and Reanimation*.

[17] Weijs P. J. M., Looijaard W. G. P. M., Dekker I. M. (2014). Low skeletal muscle area is a risk factor for mortality in mechanically ventilated critically ill patients. *Critical Care*.

[18] Moisey L. L., Mourzakis M., Cotton B. A. (2013). Skeletal muscle predicts ventilator-free days, ICU-free days, and mortality in elderly ICU patients. *Critical Care*.

[19] Kalantari K., Bolton W. K. (2013). A good reason to measure 24-hour urine creatinine excretion, but not to assess kidney function. *Clinical Journal of the American Society of Nephrology*.

